# Electrically Insulated Sensing of Respiratory Rate and Heartbeat Using Optical Fibers

**DOI:** 10.3390/s141121523

**Published:** 2014-11-14

**Authors:** Ernesto Suaste-Gómez, Daniel Hernández-Rivera, Anabel S. Sánchez-Sánchez, Elsy Villarreal-Calva

**Affiliations:** Electrical Engineering Department, Bioelectronics Section, Centro de Investigación y de Estudios Avanzados del IPN, Av. IPN 2508, Col. San Pedro Zacatenco, C.P. 07360, D.F., Mexico; E-Mails: hernandezr@cinvestav.mx (D.H.-R.); asanchezs@cinvestav.mx (A.S.S.-S.); evillareal@cinvestav.mx (E.V.-C.)

**Keywords:** fiber optics, heart rate, patient safety, photoplethysmography, respiratory rate, sensors

## Abstract

Respiratory and heart rates are among the most important physiological parameters used to monitor patients' health. It is important to design devices that can measure these parameters without risking or altering the subject's health. In this context, a novel sensing method to monitor simultaneously the heartbeat and respiratory rate signals of patients within an electrically safety environment was developed and tested. An optical fiber-based sensor was used in order to detect two optical phenomena. Photo-plethysmography and the relation between bending radius and attenuation of optical fiber were coupled through a single beam light traveling along this fiber.

## 1. Introduction

Respiratory and heart rates are essential physiological parameters to monitor patients and assess wellness in both medical studies and physical rehabilitation, since they offer important basic information about body functions. The typical heart rate in adults is 60–100 beats per minute; in contrast the respiratory rate is given as 4–50 breaths per minute under certain conditions [1].

In this context, the design and use of technological devices in medical practice involves the participation of several expert communities, including engineers, scientists, and medical doctors. Thus, to delve into what happens to the devices (designed by engineers) when used in medical practice implies not only uncovering what engineering and medical practices entail, but also to address how both agendas could converge or incorporate common goals. Regarding engineering as a discipline, the National Research Council (NRC) [2] pointed out its problem-solving approach to design and create human-made products under certain conditions or constraints. Some of those constraints involve taking into account principles that encompass science and scientific laws, budget restrictions, available materials, sustainability, electrical safety, ergonomics, and ethical issues [3].

Thus, there are many instruments that are useful in medical practice and allow us to sense vital signals; a special case of interest in this paper are respiratory rate signals where thermistors, piezoelectric devices or directly airflow meters [4,5] are the most used methods to monitor them. In another special case, the most common techniques used to obtain heartbeat signals are photo-plethysmography, impedanciometry, piezoelectric sensors, and others [6,7]. Current new technology trends emphasizes the use of multi-sensors to conveniently and simultaneously sense various vital signals, and moreover, new sensors should provide minimal interference with the environment and must require minimum care; costs should also be as low as possible [8].

Recently, medical fields have demanded the development of new sensors to perform vital signal monitoring in hazardous environments. Fiber optic sensors can solve problems involving high temperature, electromagnetic interference and humidity environments, while they have improved greatly in the electrical safety aspect [9–19].

Electrical safety is a very important topic in biomedical engineering, especially when related to patient monitoring. Patients are already ill, and as a consequence, they are more susceptible to electrical hazards. There are many protective methods to reduce the risk of electrical accidents; their complexity depends of the specific hazards in the patient area [20,21]. The use of optical fiber as sensor has well known benefits in medical applications; one of the most useful ones is that offers electrical insulation due to the fact that fiber is the only surface of the sensor in contact with the patient. The use of fiber optic sensors is also ideal for applications at non-perturbing magnetic fields allowing its use in RF, magnetic and microwave environments [22,23].

Two optical phenomena are of interest for this paper. The first one is the relationship between bending radius and light attenuation of the fiber optic. The second phenomenon occurs when light falls on human skin. During this process, light is absorbed, reflected and diffusively scattered in tissue and blood; the optical absorption of light on skin changes due to the pulsing blood. The resulting signal is commonly acquired through a system where a light beam falls on some body part (for example the forefinger or ear) and a photoreceptor collects the transmitted light. A signal conditioning stage is necessary to get a plethysmography waveform. In other cases the same process is realized using optical fiber in order to achieve a longer trajectory of light before it arrives to the electronic stage.

There are some devices that acquire heart and respiratory rates at the same time, but they require the use of complex filtering stages, high digital processing, complex and delicate hardware [24,25]. Applications in MRI or humid environments are real; therefore the use of optic fiber sensors at those situations demonstrates the suitability of these devices.

## 2. Materials and Methods

### 2.1. Sensing

**Figure 1.**
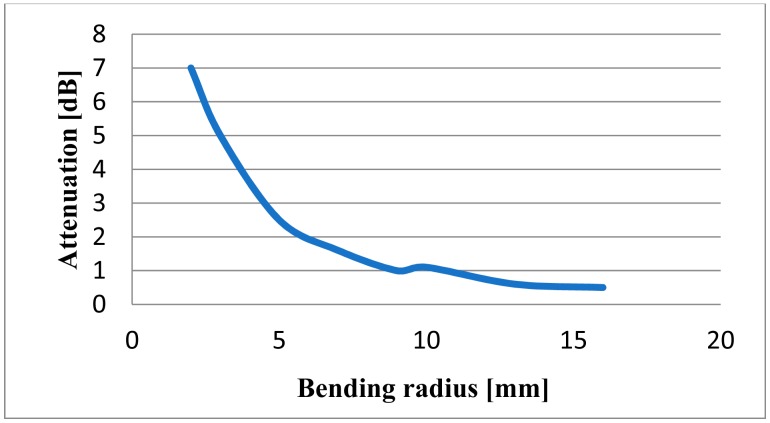
Light attenuation as a function of bending radius.

Sensing of physiological signals was realized exploiting two optical phenomena coupled in a fiber optic system. The robustness and easy-handling of a plastic optical fiber were reasons to use this material. Table 1 shows main characteristics of the fiber optic materials. In order to get the first phenomenon the optical fiber is bent; attenuation of light intensity starts at a bending radius of about 15 mm and increases roughly proportionally with a smaller bending radius. Figure 1 indicates the behavior of fiber optic to different bending radii [12]. Due to the alternating strain present at a dynamic application, it is possible to break the bending fiber limit when we approach a radius of 3 mm.

Attenuation of light intensity depends on the fiber elongation [12]; this relation is indicated in (1):

(1) $$I=cos^{2}\;(\Delta \beta \cdot \Delta l_{\text{fiber}})$$

where Δβ is the difference of the propagation constant and Δ*l_fiber_* is the fiber elongation.

**Figure 2.**
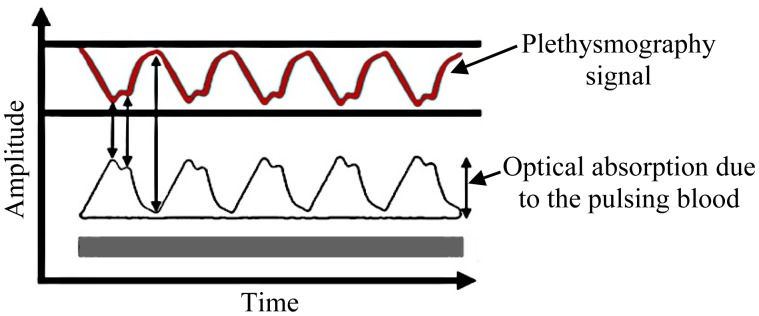
Photoplethysmography signal.

In relation to the second phenomenon, a plethysmography signal is generated due to the variation of light absorption as illustrated in Figure 2. Thus, there is a relation between the light received and the heartbeat [27], which should serve to modulate the light traveling throughout the optic fiber.

### 2.2. Proposed Design

The attenuation of light intensity that appears when the optical fiber is deformed allows building a respiratory rate sensor. The fiber should be deformed according to the chest volume in a respiration cycle. This design has been proved previously [12,14,18]. The aim of this paper is to couple the aforementioned phenomena in a single light trajectory throughout an optical fiber, in order to simultaneously obtain respiratory rate and heartbeat signals using the same device. Therefore, the design can operate in harsher environments than conventional electronics can do. On the other hand, since the only contact with the patient is the optical fiber we can assure that the user never will be exposed to any electrical hazards.

### 2.3. Design of the Sensor Device

The design method is based on placing a light emission source at one extreme of an optical fiber and a light reception device at the other end. The first stage of the development consists in polishing the optical fiber ends, since the light transmission requires clarity and an unobstructed path through its core. Polishing is needed because otherwise back reflections and other signal losses might occur. A special polishing paper was employed to meet this requirement. Once the sensor is built, there is no chance of direct contact with the fiber that could damage the polishing work.

**Figure 3.**
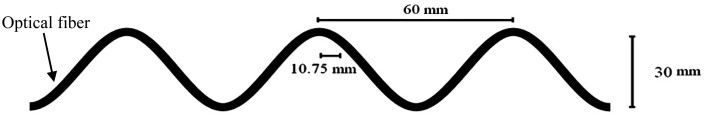
Diagram of bending radius in fiber optic.

In the next stage a fiber part was fixed on a section of an elastic strip which should be attached to the chest wall where the maximum changes during inspiratory phase are noticed. The macro-bending radius changes are expected between 10.75 mm and 13 mm while the patient exhales and inhales. A diagram of the bending radius is shown in Figure 3.

**Figure 4.**
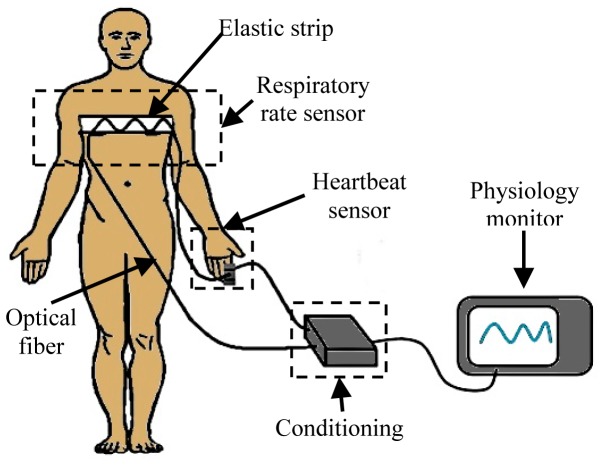
Diagram of the respiratory rate and heartbeat sensors placed on the isolated patient.

The trajectory of the fiber was set up to reach the right forefinger in order to transmit light through that tissue. The other segment of the optical fiber is placed on the other side of the same finger to receive the transmitted light. The light that travels through the optical fiber is doubly modulated. The first modulation corresponds to the respiration cycle and the other one to the heartbeat. The modulated light contains the desired information, *i.e.*, respiratory rate and heart rate. The diagram of Figure 4 shows where the optical fiber was placed according to the proposed design. As is shown, the patient will be always electrically isolated because the body has contact only with the optical fiber, and due to the nature of the material there is no possible electrical pathway.

### 2.4. Conditioning

**Figure 5.**
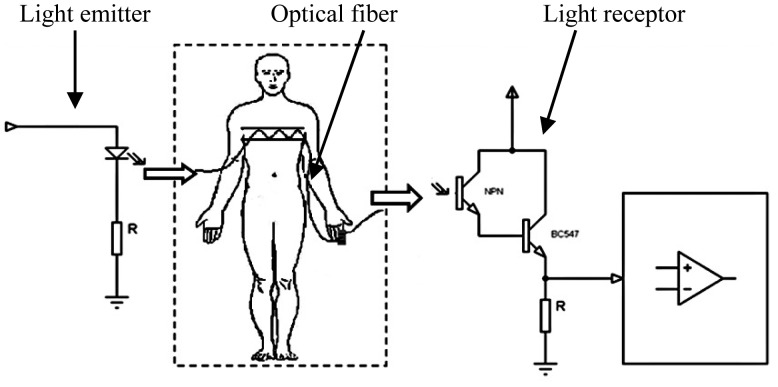
Electronic circuits used for transmission and reception of light in the fiber optic.

The light source was provided through a LED with a peak wavelength of 640 nm, coupled at one end of the optical fiber; 640 nm was chosen because this kind of optic fiber is designed to work at this wavelength and moreover it is commonly used in pulse-oximetry. The receptor was improved using a phototransistor at Darlington configuration, with the aim of amplifying the small changes of light intensity. The light that arrives to the phototransistor is enough for generate a response at the detector but, clearly a part of light is lost in the process. To know that quantity of light lost before arrival at the receptor, LED light was measured directly with a phototransistor and also using optical fiber as interface with the same phototransistor; the result shows that only 7% ± 1% of the LED light arrives at the phototransistor when optical fiber was used as interface. Figure 5 displays the electronic circuits for the transmission and reception of light.

**Figure 6.**
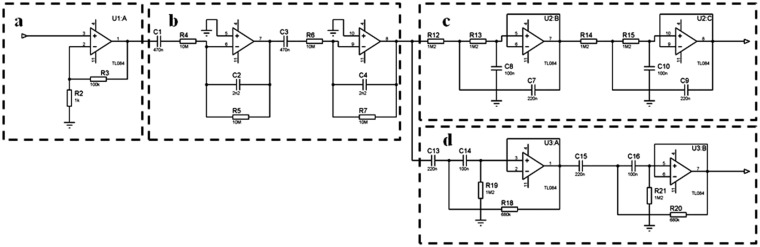
Amplifying stage (**a**), second order cascade filtering from 0.03 Hz to 7.2 Hz (**b**); second order filters in cascade for respiratory rate (**c**) and for heart rate (**d**).

The next stage consists in an analog signal conditioning process. First the signal is amplified by a factor of 100 and filtered with a second order cascade filtering (0.03 Hz to 7.2 Hz) in order to eliminate noise and interferences; after the signal is split by two bandwidths, the first bandwidth was tuned between 0.1 Hz and 1 Hz in order to get the respiratory rate signal and the second bandwidth was tuned between 1 Hz and 3.5 Hz to get the heartbeat signal. The topology chosen for the implementation of this filtering was the Sallen Key configuration using operational amplifiers. The bandwidths for the filters were elected experimentally considering the typical frequency of those signals in humans [28]. The schematic of the electric circuits is illustrated in Figure 6.

## 3. Results

**Figure 7.**
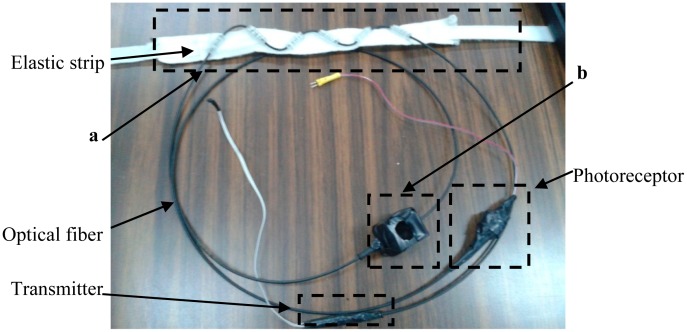
Respiratory rate (**a**) and heartbeat (**b**) sensors.

**Figure 8.**
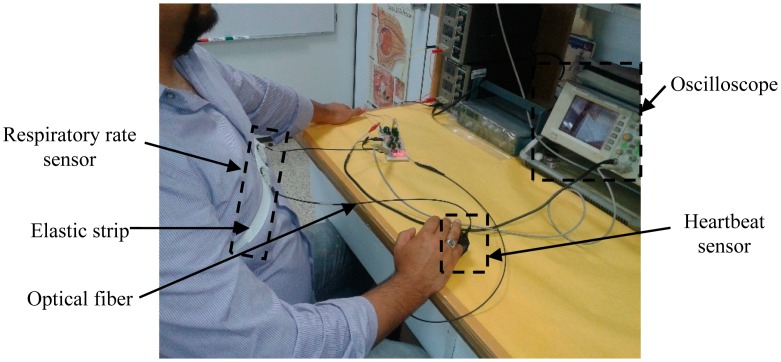
Test subject using fiber optic sensor.

Pre-testing was performed with the purpose of checking the response of the sensor and assessing the viability of the system as a respiratory rate and heartbeat sensor. Figure 7 shows the proposed fiber optic sensor and Figure 8 illustrates how the fiber optic sensor is placed on a subject test.

**Figure 9.**
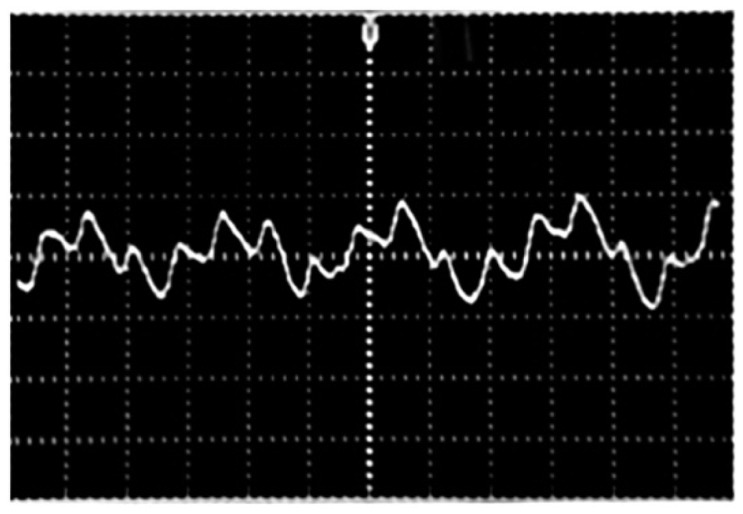
Composite signal with the respiratory rate and the heartbeat.

Figure 9 displays the received signal before the demodulation stage. Two signals are coupled in one single signal. The respiratory rate signal is slower than the heartbeat signal.

**Figure 10.**
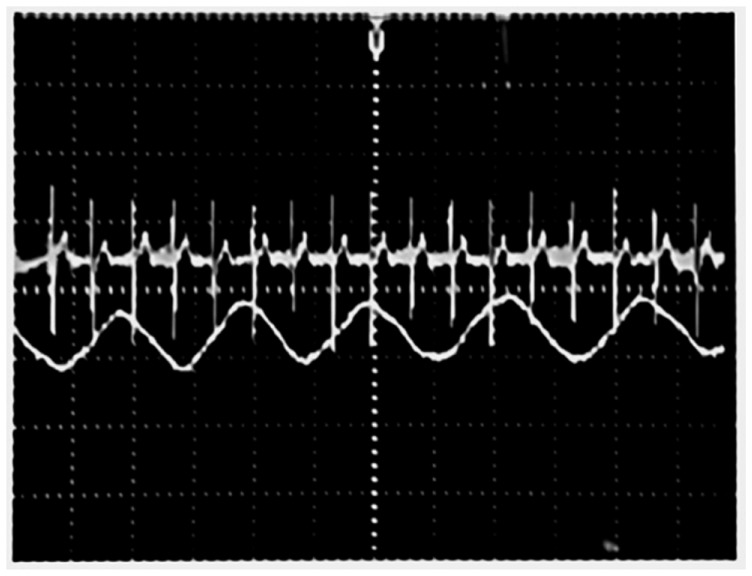
Acquired signals of respiratory rate and ECG.

**Figure 11.**
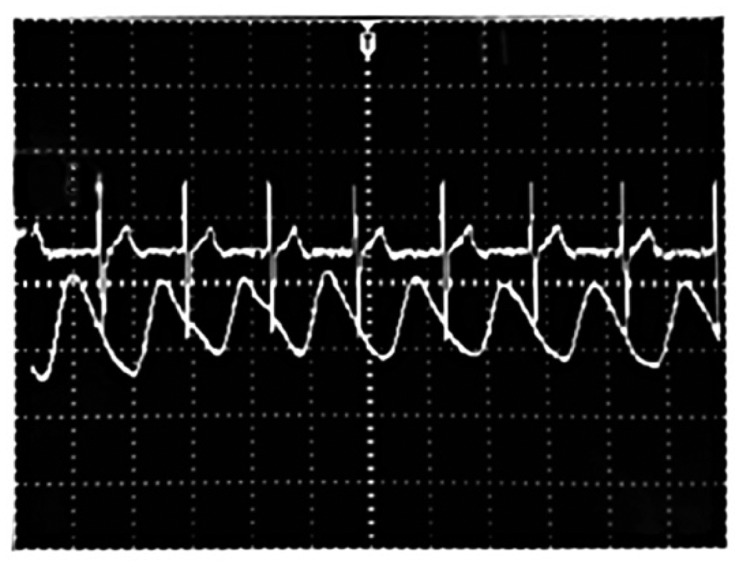
Acquired signals of heartbeat and ECG.

Once the filtering process was checked and approved, the signals of interest were acquired from 10 subjects. In order to show both signals clearly and also as a form of validation, we show simultaneously these waveforms with the corresponding ECG for one test subject. A commercial BioAmp 100 electrocardiograph device (Axon Instruments, Sunnyvale, CA, USA) was used to record the ECGs. The signals acquired are illustrated in Figures 10 and 11. The mean respiratory rate obtained was approximately 25 breaths per minute; this frequency is contrasted with the ECG signal which is faster. The plethysmography signal provided a heart rate of 75 beats per minute.

**Figure 12.**
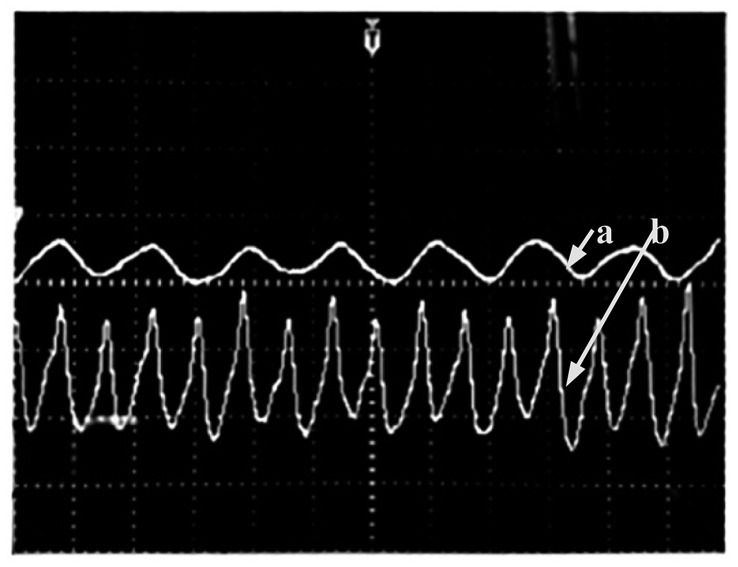
Respiratory rate (a) and heartbeat (b) signals.

Figure 12 exhibits the respiratory rate and heartbeat signals from the same subject simultaneously and clearly. As can be seen from the figure heartbeat signal is faster than the respiratory rate.

**Figure 13.**
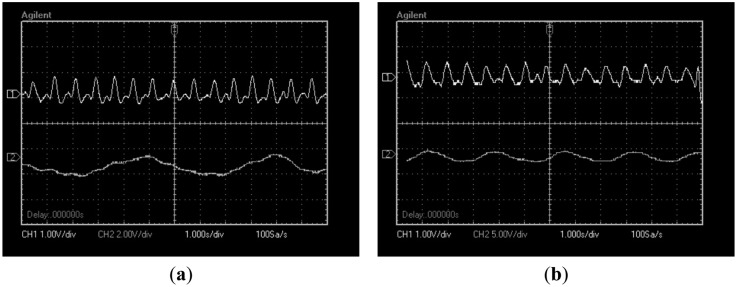

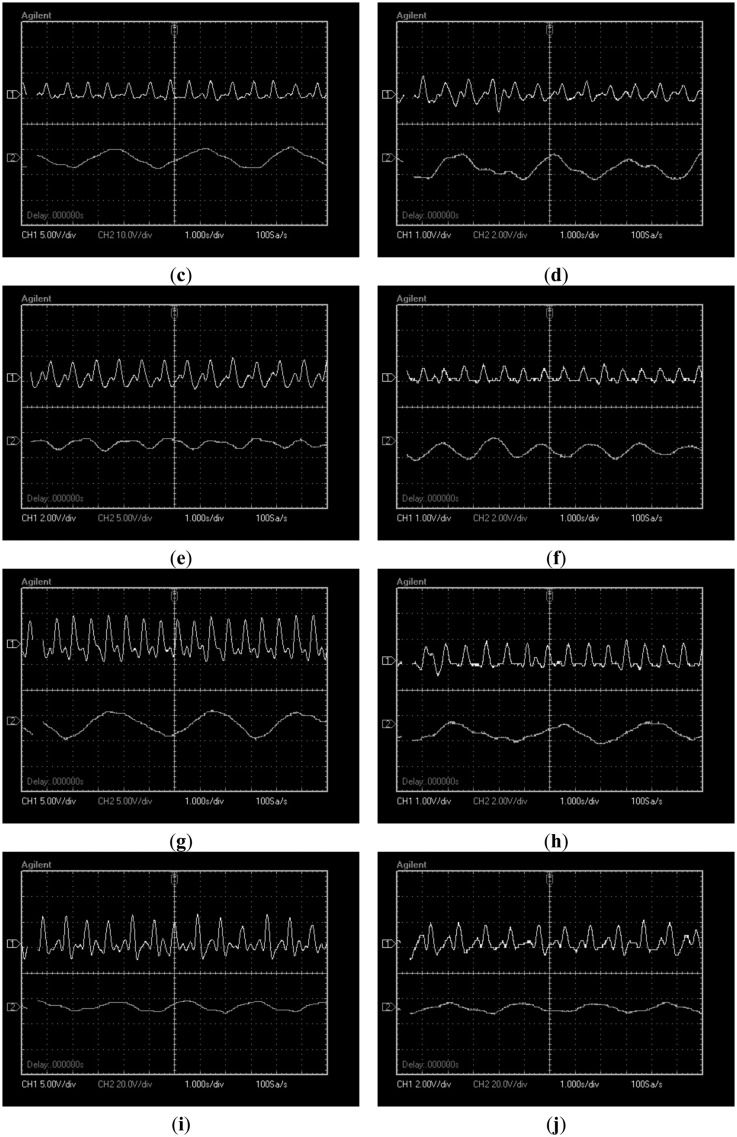
Signals obtained of proposed sensor for 10 subject tests (**a**–**j**).

Finally, Figure 13 shows signals of 10 test subjects where heart beat and respiratory signal appear at the same time. The test subjects were selected in a range from 20 to 30 years old. In the graphics variations in respiratory and heart beat rate due to physiologic differences among subjects can be seen.

## 4. Discussion

The data obtained from the subjects were analyzed. The typical bandwidths were considered and the waveforms corresponding to one subject were compared with the ECG given by a commercial device as one way to validate the viability of the sensor. The tests performed in 10 subjects show the capacity of the proposed device for simultaneous sensing of respiratory and heart beat signals. The filtering stage improved in this trial allowed us to solve the problems regarding to the coupled signal acquired by the method presented in this paper.

The purpose in this stage of the research, presented in this paper, corresponds only to the development of the sensor, including the filtering stage, where the time between peaks is a quantifiable parameter of the waveform. Analyzing the tests performed it can be observed that signals between subjects have concordance regarding the typical waveform of both signals of interest. The accuracy and stability will be of importance when a numeric value is obtained and it can be compared and validated with a commercial device, but in this moment the interest is just in the waveforms obtained by the proposed sensor.

The proposed sensor worked as expected. In future work we plan to optimize it and will corroborate the results indicated in this paper through a test involving more subjects and will do validation comparing it numerically against commercial equipment. To get the best performance of this device special care must be taken when choosing the diameter of the optical fiber; this parameter defines the maximum allowable flexion that does not damage the optical fiber. It is worth mentioning that the optic fiber polishing technique is also important for the improvement of the transmission and reception of the light.

## 5. Conclusions

The functioning of the proposed sensor should allow a vast number of applications in the field of monitoring physiological signals since it will be useful in harsher environments than those where conventional sensors can be used, such as electromagnetic environments, high temperatures, in water environments and others. Some examples of those environments are MRI, aquatic rehabilitation, physiology in diving, aviation, and space, and also we can assess the electrical safety in the patient.

Actually there are devices based on fiber optics which solve the aforementioned problems but at high cost and using bulky equipment. In this paper a method to acquire two important signals for monitoring vital signals is presented, and a device based on optic fiber which offers users versatility, comfort and involving no electrical connection to the body is developed and tested. The elements used to construct the sensor are a single optical fiber, an elastic strip, a finger enclosure and conventional analog electronic components. For these reasons and the proper functioning of the proposed sensor shown in the tests, the device offers robustness and could be easily adapted in a commercial monitoring system.

## Figures and Tables

**Table 1.** Optical fiber specifications [26].

**Characteristics**	**Values**
Core refractive index (***n*_1_**)	1.492
Clad refractive index (***n*_2_**)	1.417
Numerical aperture (***NA***)	0.47
Acceptance Angle (°)	56e
Service temperature (***T***)	–20 °C to 85 °C
Attenuation	$160enua\frac{dB}{Km}(660nm)$
Generals	Polymethylmethacrylate core, fluorinated polymer cladding
Core diameter (***d*_1_**)	1mm
Clad diameter (***d*_2_**)	2.2mm
